# Albumin and psychological resilience: key modifiable factors for hospital length of stay in ulcerative colitis

**DOI:** 10.3389/fnut.2025.1704658

**Published:** 2026-01-08

**Authors:** Man Yang, Lei Lei, Huiying An, Lili Shi, Yingya He, Meng Yang, Lifang Peng, Liming Li, Xiangming Ding, Xiaoying Luo

**Affiliations:** 1 Department of Gastroenterology, Henan Provincial People's Hospital, People's Hospital of Zhengzhou University, Zhengzhou University, Zhengzhou, China; 2 Department of Nursing, Henan Provincial People's Hospital, People's Hospital of Zhengzhou University, Zhengzhou University, Zhengzhou, China

**Keywords:** ulcerative colitis, length of stay, biopsychosocial factors, disease severity, negative binomial regression

## Abstract

**Background:**

Reducing the length of stay (LOS) for patients hospitalized with inflammatory bowel disease is crucial for lowering healthcare costs and improving patients’ quality of life. However, the determinant factors of LOS in ulcerative colitis (UC) patients are incompletely understood.

**Methods:**

Participants were obtained from the Henan Provincial People’s Hospital during the period from January 2018 to April 2025. Information on multidimensional determinants of LOS was collected using a questionnaire. Spearman correlation analysis and negative binomial regression models were performed to identify factors associated with LOS. A subgroup analysis was performed to examine the disease stage and severity in relation to LOS. The interaction of identifying factors was analyzed.

**Results:**

A total of 400 active UC-related hospitalizations were identified. Correlation analysis showed that LOS was positively correlated with mMayo score, improved Mayo endoscopic score, white blood cell count, platelet, C-reactive protein, and gamma-glutamyl transferase levels. However, LOS was negatively correlated with red blood cell count, hemoglobin, albumin, and total cholesterol levels. A negative binomial regression model showed that albumin reduction was associated with prolonged LOS in active UC patients, regardless of disease severity. Decreases in albumin level and Connor–Davidson Resilience Scale score were associated with increased LOS, and their corresponding estimated incidence rate ratios and 95% confidence intervals were 0.967 (0.937, 0.997) and 0.434 (0.188, 1.003), respectively. An interaction of these variables on LOS was observed in the remission stage of patients.

**Conclusion:**

This study found that lower serum albumin levels were significantly associated with a longer LOS in patients with active UC. Furthermore, both lower albumin levels and poorer psychological resilience were associated with prolonged LOS. This study’s findings suggest that albumin optimization may be an effective strategy for reducing LOS in active UC, and further integrating psychological resilience–building interventions into standard care could help shorten hospitalization and improve outcomes for patients in remission.

## 1 Introduction

Ulcerative colitis (UC), a subtype of inflammatory bowel disease (IBD), is characterized by non-specific, chronic progressive, and relapsing colorectal inflammation. This lifelong condition significantly impairs patients’ quality of life and poses a growing challenge to healthcare systems worldwide. Despite advancements in therapeutic treatments, including 5-aminosalicylic acid (5-ASA), corticosteroids, immunosuppressants, biologics, and small molecules for UC, the hospitalization rates continue to rise around the world, especially in newly industrialized countries such as those in Latin America and China, which reflects the persistent challenges in achieving sustained disease control and underscores the increasing burden on national economies and global healthcare systems (1–3).

The length of stay (LOS) is defined as the duration from hospital admission to discharge and serves as a sensitive metric for assessing hospital resource utilization and short-term outcomes (4, 5). The LOS among patients with UC exhibits considerable variability due to a range of complex factors. Clinical determinants include disease severity and extent, presence of comorbidities, UC-related complications (e.g., toxic megacolon, severe colorrhagia, intestinal perforation, colorectal cancer), and nutritional status. Beyond biological factors, emerging evidence also highlights the importance of therapeutic approaches, health literacy, family economic status, psychological wellbeing, and socioeconomic conditions in shaping hospitalization outcomes (6, 7). Nevertheless, the dominant predictor of LOS remains unclear, complicating prognosis estimation, resource allocation, and the development of individualized care planning.

Effective management for patients with UC requires a multidisciplinary approach that integrates pharmacological therapy, individualized nursing interventions, and social resource support. Comprehensive biopsychosocial models have been shown to improve clinical response and remission rates, enhance quality of life, and stabilize household economic costs (6, 7). Growing evidence also indicates that both abnormal clinical indicators and chronic psychological stress play important roles in triggering deterioration and relapse of IBD, which may be closely related to increased LOS (8). Additionally, family financial stress may result in delayed medical consultations, poor adherence to medications, and increased complications in patients, and these contribute to prolonged LOS in the hospital. Recent studies further highlight financial toxicity, a term describing the subjective economic burden experienced by patients, as a significant factor impairing wellbeing and a potential predictor of extended LOS (9, 10). Therefore, identifying the key modifiable factors that most significantly influence LOS is crucial for optimizing care delivery and reducing the economic burden on both healthcare systems and families.

In this study, we investigated key biopsychosocial determinants influencing LOS in patients with UC through comprehensive clinical profiling and assessments of psychological resilience, financial toxicity, self-management behaviors, and family and social support networks. Our findings will help predict LOS and support the development of personalized care pathways to reduce hospitalizations while maintaining high standards of care. Furthermore, this study aimed to identify modifiable determinants of healthcare utilization patterns, improve medical resource utilization, and optimize healthcare delivery for patients with UC, ultimately contributing to the development of effective strategies to improve hospital care efficiency and reduce unnecessary healthcare expenditures.

## 2 Methods

### 2.1 Clinical population

We conducted a single-center, retrospective study at Henan Provincial People’s Hospital from January 2018 to April 2025. Adult patients (aged 18–80 years) with a confirmed diagnosis of UC based on the modified Truelove–Witts criteria, along with endoscopic and pathologic examination (11), and a disease duration of at least 6 months were consecutively screened. Disease severity was assessed based on clinical and laboratory parameters, including stool frequency, rectal bleeding, pyrexia, tachycardia, and anemia, as well as erythrocyte sedimentation rate (ESR), C-reactive protein (CRP), and albumin levels (11). Eligibility was restricted to those experiencing their first admission. Patients with colitis resulting from other etiologies (e.g., Crohn’s disease, infective colitis, radiation-induced colitis, drug-induced colitis, and unexplained colitis) were excluded. Meanwhile, patients who were pregnant, in the lactation period, undergoing colectomy, or who had malignant tumors, hematologic diseases, mental illness, serious cardiovascular diseases or cerebrovascular diseases, or serious liver and kidney dysfunction were further excluded.

The study was registered at the Chinese Clinical Trial Registry (www.chictr.org.cn, ChiCTR1900026035) and approved by the Committee on the Ethics of Henan Provincial People’s Hospital ((2018) NO.03–01). All patients provided written informed consent before participating in this study.

### 2.2 Data collection

Upon enrollment, all patients underwent comprehensive assessments on the day of initial admission, including demographics (e.g., age, sex, blood pressure, height, weight, marital status, residence, degree of education, insurance information, household income, medical history, and duration of UC) and documentation of disease characteristics (disease extent, severity, and endoscopic score). All patients underwent laboratory testing on the day of initial admission, encompassing: (1) complete blood count and inflammation indices: white blood cell (WBC) count, red blood cell (RBC) count, hemoglobin (HB), platelet (PLT), CRP, and ESR; (2) hepatic parameters: albumin, alanine aminotransferase (ALT), aspartate aminotransferase (AST), serum alkaline phosphatase (ALP), gamma-glutamyl transferase (GGT), and AST/ALT ratio; (3) blood glucose and lipid parameters: serum glucose (GLU), total cholesterol (TC), and triglyceride (TG); and (4) renal parameters: blood urea nitrogen (BUN) and serum creatinine (SCR).

Additionally, the following validated questionnaires were administered: (1) Chinese version of the Comprehensive Score for Financial Toxicity, which contains 11 items each rated on a 5-point Likert scale (range, 0–44). Items 2, 3, 4, 5, 8, 9, and 10 were reverse-scored, and items 1, 6, 7, and 11 were positively scored, so that lower total scores indicate greater financial distress (12, 13). (2) Chinese version of the Patient Health Questionnaire-9 (PHQ-9), used for screening the presence and severity of depression, which contains nine items each rated on a 4-point Likert scale (range, 0–27) (14). A positive scoring direction was applied to all items so that higher scores reflect more severe depression symptoms. (3) Chinese version of the Family Communication Scale, which contains nine items each rated on a 4-point Likert scale (range, 9–36). The scale primarily assesses family communication quality and family support in patient–family interactions (15). Items 2, 4, 6, 8, and 9 were reverse-scored, and items 1, 3, 5, and 7 were positively scored, so that higher scores indicate better family communication. (4) Chinese version of the Connor–Davidson Resilience Scale, a 10-item questionnaire with each item rated on a 5-point Likert scale (range, 10–50). All items were positively scored, with higher scores indicating stronger psychological resilience (16). (5) Chinese version of the IBD Self-Management Behavior Scale, a 32-item questionnaire with each item rated on a 5-point Likert scale (range, 32–160), which timely identifies the behavioral weaknesses of patients with IBD, contributing to precisely intervening in these behaviors and predicting health outcomes in patients with IBD (17). All items were positively scored, with higher scores indicating greater self-management capacity. (6) Chinese version of the Social Support Scale containing 10 items. These items help identify factors that influence psychological stress in patients with UC (18). All items were scored in the positive direction to denote stronger social support and higher total scores. (7) Chinese version of the Utrecht Work Engagement Scale (UWES) containing nine items, each rated on a 7-point Likert scale (range, 9–63). The scale evaluates patients’ occupational psychology (19). All items were scored in the positive direction to indicate greater work engagement. The questionnaires were examined and demonstrated good internal consistency. The results showed that the Cronbach’s *α* values of the Financial Toxicity, PHQ-9, Family Communication Scale, Connor–Davidson Resilience Scale, Self-Management Behavior Scale, Social Support Scale, and UWES items were 0.96, 0.86, 0.72, 0.93, 0.89, 0.62, and 0.99, respectively, indicating moderate-to-high internal consistency. The details of the questionnaires are provided in the Supplementary material.

### 2.3 Follow-up

Enrolled patients were followed up until discharge, at which point they had achieved clinical stability or a clinical response to treatments. The LOS in our study was defined as the duration from hospital admission to discharge. We recorded LOS and the therapeutic approaches of each patient, including 5-ASA, corticosteroids, immunosuppressants, biologics, and small molecules.

### 2.4 Statistical analysis

Both missing data and outliers were minimal, at less than 1% in this study. Missing values were imputed using the mode and mean for categorical and continuous variables, respectively. Measurement data following a normal distribution are presented as mean ± standard deviation (SD). Data following a non-normal distribution are presented as median (interquartile range (IQR)). Spearman correlation analysis was used to analyze the relationship between each pair of variables. Linear regression models were used to explore factors influencing albumin levels among patients with UC at different stages, and the estimated β values with 95% confidence intervals (CIs) were calculated to assess the effect sizes of the factors on albumin levels.

The study’s dependent variable, LOS, was treated as a count variable, ranging from 1 to 56 days. The mean and variance of LOS in this study were 10.08 and 50.14 days, respectively. A negative binomial regression model was employed to examine the factors associated with LOS, due to the skewness and overdispersion of LOS (20), where the variance exceeded the mean (21). First, bivariate negative binomial regression models and Spearman correlation analyses were initially conducted to identify the factors associated with LOS. Second, variables that were significant in the bivariate analysis were subsequently entered into a multivariable model using a forward selection approach. To identify the best-fitting model, the final multivariable model was selected based on the lowest Akaike Information Criterion value (22). The findings of this study reported incidence rate ratios (IRRs) and 95% CIs. Moreover, the interaction between the Connor–Davidson Resilience Scale score and albumin on LOS was evaluated among UC patients in remission. All data analysis was performed using SPSS 21.0 (SPSS Institute, Chicago) and R 4.1.1. Statistical significance was assigned at a two-sided *p* < 0.05.

## 3 Results

### 3.1 The clinical and biopsychosocial characteristics of patients with UC

**Figure 1:**
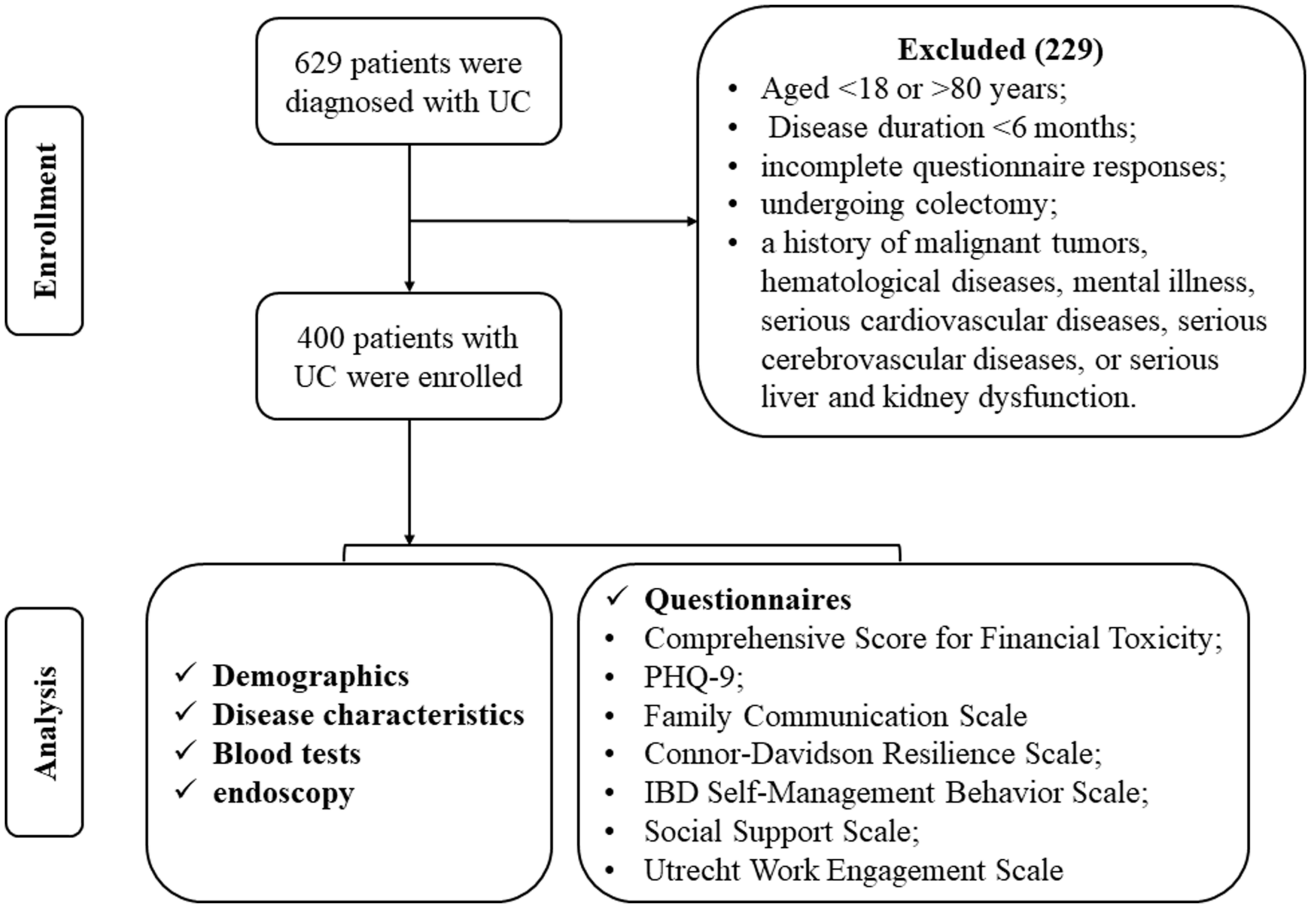
Flowchart of the clinical cohort of enrolled patients with UC.

Among the 629 initially identified UC patients, 229 patients were excluded due to being aged <18 or >80 years, having a disease duration of less than 6 months, incomplete questionnaire responses, undergoing colectomy, or having a history of malignant tumors, hematologic diseases, mental illness, serious cardiovascular diseases, serious cerebrovascular diseases, or serious liver and kidney dysfunction. Consequently, 400 patients with UC were included in the final analysis (Figure 1).

As summarized in Table 1, the median age of patients was 50 years (interquartile range (IQR): 37–58). A total of 244 patients (61%) were men and 156 patients (39%) were women. The majority of patients were married (*n* = 371, 92.75%). Although 72.75% of patients had an educational level of secondary school or less and more than half of patients (58.75%) resided in rural areas, 98% of patients were covered by China’s medical insurance system, either through Employee Basic Medical Insurance (EBMI) or Urban–Rural Resident Basic Medical Insurance (URRBMI). The median Comprehensive Score for Financial Toxicity, Family Communication Scale score, and Social Support Scale score were 17 (IQR: 6–24), 20 (IQR: 19–22), and 28 (IQR: 2–35), respectively.

**Table 1:** Summary of demographics, disease features, financial toxicity, and biopsychosocial characteristics of the enrolled patients with UC.

Characteristics	Patients (*n* = 400)
Age (in years, median (IQR))	50 (37, 58)
Sex, *n* (%)	
Male	244 (61%)
Female	156 (39%)
Marital status, *n* (%)	
Single	26 (6.50%)
Married	371 (92.75%)
Separated	1 (0.25%)
Widowed	2 (0.50%)
Degree of education, *n* (%)	
Primary education or less	136 (34.00%)
Secondary education	155 (38.75%)
Tertiary education or higher	109 (27.25%)
Domicile, *n* (%)	
Urban area	165 (41.25%)
Rural area	235 (58.75%)
Insurance information, *n* (%)	
EBMI	149 (37.25%)
URRBMI	243 (60.75%)
NRCMS	3 (0.75%)
Out-of-pocket payment	5 (1.25%)
Smoking, *n* (%)	
Never smoked	311 (77.75%)
Former smoker	51 (12.75%)
Current smoker	38 (9.50%)
Alcohol use, *n* (%)	
Never drank	302 (75.5%)
Former drinker	44 (11.0%)
Current drinker	54 (13.5%)
History of other diseases	
Hypertension, *n* (%)	39 (9.75%)
Coronary heart disease, *n* (%)	7 (1.75%)
Diabetes, *n* (%)	21 (5.25%)
Cerebrovascular disease, *n* (%)	15 (3.75%)
Duration of UC (years, median (IQR))	3.0 (1.0, 6.8)
Disease staging, *n* (%)	
Remission stage	34 (8.5%)
Active stage	366 (91.5%)
Disease severity among patients with active UC (*n* = 366), *n* (%)	
Mild	58 (14.50%)
Moderate	231 (57.75%)
Severe	77 (19.25%)
Disease extent, *n* (%)	
Proctitis	39 (9.75%)
Left-sided colitis	144 (36.00%)
Pancolitis	217 (54.25%)
mMayo score (median (IQR))	8 (6, 9)
IMES (median (IQR))	2 (2, 3)
BMI (kg/m^2^, mean±SD)	23.2 ± 2.7
Complete blood count and inflammation indices	
WBC (×10^9^/L, median (IQR))	6.35 (5.10, 8.41)
RBC (×10^12^/L, median (IQR))	4.21 (3.73, 4.63)
HB (g/L, median (IQR))	118.0 (98.0, 136.0)
PLT (×10^9^/L, median (IQR))	277 (217, 359)
CRP (mg/L, median (IQR))	4.55 (0.68, 26.54)
ESR (mm/h, median (IQR))	24.0 (12.0, 46.0)
Hepatic parameters	
ALB (g/L, mean±SD)	36.7 ± 7.2
ALT (U/L, median (IQR))	14.7 (10.2, 22.3)
AST (U/L, median (IQR))	16.6 (13.0, 22.1)
AST/ALT ratio	1.16 (0.91, 1.46)
ALP (U/L, median (IQR))	68.6 (58, 84.6)
GGT (U/L, median (IQR))	17.4 (12.5, 25.3)
Blood glucose and lipid parameters	
GLU (mmol/L, median (IQR))	4.6 (4.1, 5.2)
TC (mmol/L, median (IQR))	3.9 (3.3, 4.6)
TG (mmol/L, median (IQR))	1.1 (0.9, 1.5)
Renal parameters	
BUN (mmol/L, median (IQR))	4.2 (3.2, 5.2)
SCR (μmol/L, mean±SD)	58.9 ± 14.3
Assessment of scales	
Comprehensive Score for Financial Toxicity (median (IQR))	17 (6, 24)
PHQ-9 score (median (IQR))	0 (0, 3)
Family Communication Scale score (median (IQR))	20 (19, 22)
Connor–Davidson Resilience Scale score (median (IQR))	28 (24, 32)
Self-Management Behavior Scale score (mean±SD)	124 ± 12
Social Support Scale score (median (IQR))	28 (23, 35)
Utrecht Work Engagement Scale score (median (IQR))	27 (0, 38)
Length of stay (days, median (IQR))	9 (5, 13)
Treatments, *n* (%)	
5-ASA alone	223 (55.75%)
5-ASA + Corticosteroids	89 (22.25%)
5-ASA + Corticosteroids+Immunosuppressant	3 (0.75%)
Corticosteroids+ Biologics	24 (6.00%)
Biologics	59 (14.75%)
Other treatments (e.g., small molecules)	2 (0.50%)

SD: standard deviation; EBMI, Employee Basic Medical Insurance; URRBMI, Urban–Rural Resident Basic Medical Insurance; NRCMS, New Rural Cooperative Medical Scheme; UC, ulcerative colitis; BMI, body mass index; mMayo, modified Mayo score; IMES, improved Mayo endoscopic score; WBC, white blood cell count; RBC, red blood cell count; HB, hemoglobin; PLT, platelet; CRP, C-reactive protein; ESR, erythrocyte sedimentation rate; ALB, albumin; ALT, alanine aminotransferase; AST, aspartate aminotransferase; ALP, alkaline phosphatase; GGT, gamma-glutamyl transferase; GLU, serum glucose; TC, total cholesterol; TG, triglyceride; BUN, blood urea nitrogen; SCR, serum creatinine; PHQ-9, Patient Health Questionnaire-9; 5-ASA, 5-aminosalicylic acid.

The median disease duration and LOS were 3 years (IQR: 1.0–6.6) and 9 days (IQR: 5–13), respectively. Among the patients, 34 patients (8.5%) in remission were hospitalized primarily for standardized maintenance therapy (e.g., biologic agents). The remaining 366 patients (91.5%) had active UC, including 58 patients with mildly active UC, 231 patients with moderately active UC, and 77 patients with severely active UC. The median Patient Health Questionnaire-9 (PHQ-9) score, Connor–Davidson Resilience Scale score, and UWES score were 0 (IQR: 0–3), 28 (IQR: 24–32), and 27 (IQR: 0–38), respectively. The mean score on the Self-Management Behavior Scale, with a standard deviation of 12, was 124 ± 12.

### 3.2 The disease staging, severity, and extent could directly affect LOS in patients with UC

As expected, the median LOS was significantly longer in the active disease group than in the remission group (9 *vs.* 5 days, *p* < 0.05). Among patients with active UC, those with severe disease had the longest LOS relative to those with mild or moderate activity (*p* < 0.05) (Tables 2, 3). The bivariate analysis showed that patients with active disease and disease severity (mild, moderate, and severe) were significantly associated with prolonged LOS relative to their corresponding counterparts, and their corresponding estimated IRR and 95% CIs were 1.65 (1.3, 2.094), 1.388 (1.152, 1.673), and 1.708 (1.376, 2.120), respectively. Similarly, patients with greater disease extent were associated with increased LOS, and the corresponding estimated IRR and 95% CI for left-sided colitis and pancolitis were 1.277 (1.011, 1.614) and 1.490 (1.189, 1.866), respectively, compared with patients with proctitis (Table 4). These results indicate that both disease severity and extent directly contribute to extended hospital stays, underscoring the urgent need for effective therapeutic strategies to achieve clinical remission and mitigate the resulting economic burden on families and society.

**Table 2:** The demographics, disease features, financial toxicity, and psychosocial characteristics of patients in the active and remission groups.

Characteristics	Disease staging		*p*-value
	Active (*n* = 366)	Remission (*n* = 34)	
Length of stay (days, median (IQR))	9 (5, 13)	5 (3, 9)	0.000^**c**^
Sex, *n* (%)			0.314^a^
Male	226 (61.7%)	18 (52.9%)	
Female	140 (38.3%)	16 (47.1%)	
Age (in years, median (IQR))	49 (37, 58)	53 (44, 58)	0.344^c^
Duration of UC (years, median (IQR))	3.0 (1.0, 6.1)	3.1 (1.2, 7.0)	0.525^c^
Disease extent, *n* (%)			0.000^**a**^
Proctitis	27 (7.4%)	12 (35.3%)	
Left-sided colitis	132 (36.1%)	12 (35.3%)	
Pancolitis	207 (56.6%)	10 (29.4%)	
mMayo score (median (IQR))	8 (6, 9)	1 (1, 2)	0.000^**c**^
IMES (median (IQR))	2 (2, 3)	0 (0, 1)	0.000^**c**^
BMI (kg/m^2^, mean±SD)	23.2 ± 2.7	23.3 ± 2.2	0.825^b^
Complete blood count and inflammation indices			
WBC (×10^9^/L, median (IQR))	6.51 (5.17, 8.60)	5.61 (4.58, 7.05)	0.023^**c**^
RBC (×10^12^/L, median (IQR))	4.20 (3.70, 4.60)	4.53 (4.15, 4.87)	0.004^**c**^
HB (g/L, median (IQR))	117 (96, 134)	136 (121, 143)	0.000^**c**^
PLT (×10^9^/L, median (IQR))	286 (225, 363)	201 (153, 242)	0.000^**c**^
CRP (mg/L, median (IQR))	6.21 (1.10, 29.27)	0.50 (0.40, 2.10)	0.000^**c**^
ESR (mm/h, median (IQR))	24.0 (13.0, 45.0)	17.0 (3.0, 47.0)	0.207^c^
Hepatic parameters			
ALB (g/L, mean±SD)	36.3 ± 7.1	41.5 ± 5.7	0.000^**b**^
ALT (U/L, median (IQR))	14.4 (10.0, 22.0)	17.6 (13.0, 32.0)	0.010^**c**^
AST (U/L, median (IQR))	16.1 (13.0, 21.6)	19.7 (15.2, 25.4)	0.003^**c**^
AST/ALT ratio	1.17 (0.91, 1.46)	1.10 (0.78, 1.40)	0.181^c^
ALP (U/L, median (IQR))	68.1 (58.1, 84.0)	72.0 (58.0, 88.7)	0.510^c^
GGT (U/L, median (IQR))	17.3 (12.5, 25.4)	18.8 (11.9, 25.0)	0.980^c^
Blood glucose and lipid parameters			
GLU (mmol/L, median (IQR))	4.60 (4.14, 5.21)	4.83 (4.20, 5.34)	0.564^c^
TC (mmol/L, median (IQR))	3.84 (3.24, 4.54)	4.20 (3.93, 5.11)	0.019^**c**^
TG (mmol/L, median (IQR))	1.12 (0.88, 1.46)	1.10 (0.90, 1.56)	0.615^c^
Renal parameters			
BUN (mmol/L, median (IQR))	4.13 (3.14, 5.22)	4.72 (4.10, 5.23)	0.042^**c**^
SCR (μmol/L, mean±SD)	58.8 ± 14.7	59.3 ± 9.1	0.785^b^
Assessment of scales			
Comprehensive Score for Financial Toxicity (median (IQR))	17 (5, 24)	17 (6, 22)	0.759^c^
PHQ-9 score (median (IQR))	0 (0, 3)	2 (0, 4)	0.139^c^
Family Communication Scale score (median (IQR))	20 (19, 22)	20 (19, 22)	0.958^c^
Connor–Davidson Resilience Scale score (median (IQR))	28 (25, 33)	27 (22, 31)	0.026^**c**^
Self-Management Behavior Scale score (mean±SD)	124 ± 12	122 ± 11	0.439^b^
Social Support Scale score (median (IQR))	28 (23, 35)	28 (23, 35)	0.886^c^
Utrecht Work Engagement Scale score (median (IQR))	27 (0, 38)	29 (0, 39)	0.565^c^

a Chi-square test was used to compare categorical variables between the two groups.

b Student’s *t*-test was used to compare continuous variables between the two groups.

c Mann–Whitney U test was used to compare non-normally distributed continuous variables between the two groups.

SD, standard deviation; UC, ulcerative colitis; BMI, body mass index; mMayo, modified Mayo score; IMES, improved Mayo endoscopic score; WBC, white blood cell count; RBC, red blood cell count; HB, hemoglobin; PLT, platelet; CRP, C-reactive protein; ESR, erythrocyte sedimentation rate; ALB, albumin; ALT, alanine aminotransferase; AST, aspartate aminotransferase; ALP, alkaline phosphatase; GGT, gamma-glutamyl transferase; GLU, serum glucose; TC, total cholesterol; TG, triglyceride; BUN, blood urea nitrogen; SCR, serum creatinine; PHQ-9, Patient Health Questionnaire-9.

**Table 3:** The demographics, disease features, financial toxicity, and psychosocial characteristics of patients with active UC.

Characteristics	Disease severity			*p*-value
	Mild (*n* = 58)	Moderate (*n* = 231)	Severe (*n* = 77)	
Length of stay (days, median (IQR))	7 (4, 9)	9 (5, 14)^*^	12 (7, 17)^*#^	0.000^**c**^
Sex, *n* (%)				0.794^a^
Male	36 (62.1%)	145 (62.8%)	45 (58.4%)	
Female	22 (37.9%)	86 (37.2%)	32 (41.6%)	
Age (in years, median (IQR))	49 (37, 56)	49 (36, 57)	51 (39, 59)	0.469^c^
Duration of UC (years, median (IQR))	2.1 (1.0, 5.0)	3.0 (1.0, 6.1)	3.0 (1.1, 7.0)	0.419^c^
Disease extent, n (%)				0.000^**a**^
Proctitis	12 (20.7%)	14 (6.1%)	1 (1.3%)	
Left-sided colitis	22 (37.9%)	91 (39.4%)	19 (24.7%)	
Pancolitis	24 (41.4%)	126 (54.5%)	57 (74.0%)	
mMayo score (median (IQR))	5 (4, 5)	8 (7, 9)^*^	11 (11, 11)^*#^	0.000^**c**^
IMES (median (IQR))	2 (1, 2)	2 (2, 3)^*^	3 (3, 3)^*#^	0.000^**c**^
BMI (kg/m^2^, mean±SD)	23.6 ± 2.4	23.3 ± 2.7	22.7 ± 3.0	0.086^b^
Complete blood count and inflammation indices				
WBC (×10^9^/L, median (IQR))	6.08 (4.88, 7.63)	6.47 (5.15, 8.60)	6.83 (5.57, 9.91)	0.139^c^
RBC (×10^12^/L, median (IQR))	4.39 (3.84, 4.75)	4.20 (3.72, 4.66)	4.08 (3.55, 4.39)^*#^	0.003^**c**^
HB (g/L, median (IQR))	126 (107, 139)	117 (96, 136)	110 (84, 126)^*#^	0.001^**c**^
PLT (×10^9^/L, median (IQR))	250 (218, 303)	286 (215, 373)^*^	315 (270, 386)^*^	0.003^**c**^
CRP (mg/L, median (IQR))	2.76 (0.50, 18.18)	5.82 (1.00, 26.43)	15.84 (2.74, 52.30)^*#^	0.001^**c**^
ESR (mm/h, median (IQR))	22 (9, 33)	22 (12, 39)	31 (16, 62)^*#^	0.016^**c**^
Hepatic parameters				
ALB (g/L, mean±SD)	37.6 ± 8.6	36.9 ± 6.9	33.3 ± 5.7^*#^	0.000^**b**^
ALT (U/L, median (IQR))	15.2 (10.3, 22.0)	14.6 (10.3, 22.0)	13.0 (8.8, 21.4)	0.360^c^
AST (U/L, median (IQR))	17.8 (14.4, 22.4)	16.7 (13.3, 22.0)	13.6 (11.1, 19.6)^*#^	0.004^**c**^
AST/ALT ratio	1.20 (0.98, 1.58)	1.17 (0.93, 1.46)	1.16 (0.83, 1.45)	0.652^c^
ALP (U/L, median (IQR))	74.1 (60.0, 85.5)	68.5 (58.6, 85.0)	67.3 (55.0, 77.4)	0.231^c^
GGT (U/L, median (IQR))	15.6 (12.9, 22.3)	17.3 (12.0, 25.4)	18.1 (13.5, 29.6)	0.331^c^
Blood glucose and lipid parameters				
GLU (mmol/L, median (IQR))	4.55 (4.18, 5.04)	4.63 (4.17, 5.29)	4.56 (4.07, 5.20)	0.730^c^
TC (mmol/L, median (IQR))	4.26 (3.44, 4.76)	3.75 (3.20, 4.48)^*^	3.84 (3.00, 4.54)^*^	0.021^**c**^
TG (mmol/L, median (IQR))	1.08 (0.79, 1.53)	1.15 (0.89, 1.46)	1.07 (0.90, 1.37)	0.722^c^
Renal parameters				
BUN (mmol/L, median (IQR))	4.51 (3.62, 5.35)	4.19 (3.17, 5.10)	3.80 (2.81, 4.97)	0.087
SCR (μmol/L, mean±SD)	59.0 ± 13.0	59.0 ± 14.5	58.3 ± 16.8	0.942^b^
Assessment of scales (median, IQR)				
Comprehensive Score for Financial Toxicity (median (IQR))	18 (6, 24)	18 (7, 24)	14 (3, 25)	0.601^c^
PHQ-9 score (median (IQR))	0 (0, 2)	0 (0, 3)	1 (0, 3)	0.122^c^
Family Communication Scale score (median (IQR))	20 (19, 23)	20 (19, 22)	20 (18, 22)	0.611^c^
Connor–Davidson Resilience Scale score (median (IQR))	27 (25, 33)	28 (24, 33)	29 (25, 32)	0.749^c^
Self-Management Behavior Scale score (mean±SD)	124.5 ± 11.8	122.8 ± 12.5	127.3 ± 11.0^**#**^	0.020^**b**^
Social Support Scale score (median (IQR))	29 (22, 36)	28 (23, 35)	27 (22, 35)	0.724^c^
Utrecht Work Engagement Scale score (median (IQR))	30 (0, 38)	27 (0, 38)	23 (0, 33)	0.340^c^

a Chi-square test was used to compare categorical variables among the three groups.

b Analysis of variance (ANOVA) was used to compare the mean difference of variables among the three groups.

c Kruskal–Wallis test was used to compare non-normally continuous variables among the three groups.

^*^p < 0.05 versus the mild group; ^#^*p* < 0.05 versus the moderate group. SD, standard deviation; UC, ulcerative colitis; BMI, body mass index; mMayo, modified Mayo score; IMES, improved Mayo endoscopic score; WBC, white blood cell count; RBC, red blood cell count; HB, hemoglobin; PLT, platelet; CRP, C-reactive protein; ESR, erythrocyte sedimentation rate; ALB, albumin; ALT, alanine aminotransferase; AST, aspartate aminotransferase; ALP, alkaline phosphatase; GGT, gamma-glutamyl transferase; GLU, serum glucose; TC, total cholesterol; TG, triglyceride; BUN, blood urea nitrogen; SCR, serum creatinine; PHQ-9, Patient Health Questionnaire-9.

**Table 4:** Variables independently associated with length of hospital stay among IBD inpatients.

Variables	Bivariate analysis (IRR, 95% CI)	*p*-value	Multivariable analysis (IRR, 95% CI)	*p*-value
Age (years)	1.002 (0.997, 1.006)	0.516		
Sex, n (%)		0.018		0.004
Male	Reference		Reference	
Female	0.855 (0.75, 0.973)		0.84 (0.746, 0.946)	
Marital status, n (%)		0.516		
Single/Separated/Widowed	1.084 (0.85, 1.382)			
Married	Reference			
Degree of education, *n* (%)				
Primary education or less	Reference			
Secondary education	1.032 (0.889, 1.197)	0.681		
Tertiary education or higher	0.911 (0.773, 1.073)	0.263		
Domicile, n (%)		0.034		
Urban area	1.149 (1.011, 1.308)			
Rural area	Reference			
Smoking, *n* (%)				
Never smoked	Reference			
Former smoker	1.149 (0.951, 1.389)	0.151		
Current smoker	1.146 (0.924, 1.421)	0.216		
Alcohol use, *n* (%)				
Never drank	Reference			
Former drinker	1.067 (0.87, 1.308)	0.536		
Current drinker	1.092 (0.906, 1.316)	0.355		
Duration of UC (years)	0.997 (0.985, 1.008)	0.557		
Disease staging, n (%)		0.000		
Remission stage	Reference			
Active stage	1.65 (1.3, 2.094)			
Disease severity among patients with active UC (*n* = 366), *n* (%)				
Mild	Reference			
Moderate	1.388 (1.152, 1.673)	0.000		
Severe	1.708 (1.376, 2.120)	0.000		
Disease extent, *n* (%)				
Proctitis	Reference			
Left-sided colitis	1.277 (1.011, 1.614)	0.041		
Pancolitis	1.490 (1.189, 1.866)	0.001		
mMayo score (median (IQR))	1.076 (1.053, 1.099)	0.000	1.045(1.023, 1.067)	0.000
IMES (median (IQR))	1.303 (1.206, 1.408)	0.000		
BMI (kg/m^2^, mean±SD)	1.014 (0.990, 1.039)	0.248		
Blood tests				
WBC (×10^9^/L)	1.03 (1.008, 1.052)	0.007		
RBC (×10^12^/L)	0.462 (0.356, 0.600)	0.000	0.633(0.493, 0.813)	0.000
HB (g/L)	0.993 (0.99, 0.995)	0.000		
PLT (×10^9^/L)	1.001 (1.000, 1.001)	0.006		
CRP (mg/L)	1.005 (1.003, 1.006)	0.000	1.002(1.000, 1.003)	0.014
ESR (mm/h)	1.002 (0.999, 1.005)	0.168		
ALB (g/L)	0.964 (0.956, 0.972)	0.000	0.982(0.972, 0.991)	0.000
ALT (U/L)	0.998 (0.994, 1.002)	0.290		
AST (U/L)	0.992 (0.985, 0.999)	0.022		
AST/ALT	0.932 (0.833, 1.044)	0.227		
ALP (U/L)	0.998 (0.995, 1.000)	0.096		
GGT (U/L)	1.002 (1.000, 1.004)	0.073		
GLU (mmol/L)	1.002 (0.966, 1.038)	0.926		
TC (mmol/L)	1.002 (1.000, 1.004)	0.059		
TG (mmol/L)	0.963 (0.866, 1.072)	0.491		
SCR (μmol/L)	0.997 (0.993, 1.002)	0.257		
BUN	0.995 (0.991, 1.000)	0.056		
Assessment of scales				
Comprehensive Score for Financial Toxicity	1.005 (0.999, 1.011)	0.140		
PHQ-9 score	1.008 (0.985, 1.031)	0.488		
Family Communication Scale score	0.999 (0.976, 1.023)	0.942		
Connor–Davidson Resilience Scale score	0.994 (0.983, 1.004)	0.225		
Self-Management Behavior Scale score	1.003 (0.997, 1.008)	0.316		
Social Support Scale score	0.999 (0.991, 1.007)	0.813		
Utrecht Work Engagement Scale score	0.996 (0.993, 1.000)	0.042	0.997(0.994, 1.000)	0.063

Disease severity includes the mild, moderate, and severe groups. Disease extent includes the proctitis, left-sided colitis, and pancolitis groups. UC, ulcerative colitis; BMI, body mass index; mMayo, modified Mayo score; IMES, improved Mayo endoscopic score; WBC, white blood cell count; RBC, red blood cell count; HB, hemoglobin; PLT, platelet; CRP, C-reactive protein; ESR, erythrocyte sedimentation rate; ALB, albumin; ALT, alanine aminotransferase; AST, aspartate aminotransferase; ALP, alkaline phosphatase; GGT, gamma-glutamyl transferase; GLU, serum glucose; TC, total cholesterol; TG, triglyceride; BUN, blood urea nitrogen; SCR, serum creatinine; PHQ-9, Patient Health Questionnaire-9; IRR, incidence rate ratio; 95% CI, 95% confidence interval.

### 3.3 The biopsychosocial characteristics of patients with UC in active and remission stages

No significant differences in sex, age, or disease duration were observed between the active and remission groups, and similar findings were observed across the mild, moderate, and severe subgroups of active UC (Tables 2, 3). As shown in Table 2, compared with the remission group, the active group had a higher proportion of patients with pancolitis, as well as significantly higher median values for the improved Mayo endoscopic score (IMES), mMayo score, WBC count, PLT count, and CRP level (all *p* < 0.05). Conversely, the active group showed lower medians for RBC count and hemoglobin level (all *p* < 0.05). Furthermore, among patients with active UC, the severe subgroup demonstrated the highest proportion of pancolitis, along with the highest median of IMES, mMayo score, PLT count, CRP, and ESR levels (all *p* < 0.05) (Table 3). Conversely, patients with severe UC had the lowest median of RBC count and hemoglobin level (all *p* < 0.05). Additionally, despite normal hepatic and renal parameters in patients with UC, patients in the active group exhibited a lower mean level of albumin and lower median levels of ALT, AST, TC, and BUN than the remission group (all *p* < 0.05) (Table 2). Moreover, among the patients with active UC, the severe group demonstrated the lowest mean level of albumin and the lowest median level of AST (all *p* < 0.05) (Table 3). These results suggested an insufficiency of liver metabolic function and urea synthesis in patients with active UC, especially in severe cases, reflecting a state of malnutrition and increased metabolic consumption.

Notably, patients in the active group exhibited a higher median score on the Connor–Davidson Resilience Scale than the remission group (*p* < 0.05) (Table 2). Moreover, the severe group had the highest mean Self-Management Behavior Scale score among patients with active UC (*p* < 0.05) (Table 3), suggesting that patients with active UC exhibit greater psychological resilience and more robust adaptive coping mechanisms when confronting UC progression.

Those findings indicate that the increased LOS may be closely linked to UC disease severity and that patients with advanced disease severity exhibit enhanced psychological resilience and self-management behaviors despite worse clinical and inflammatory indicators.

### 3.4 Clinical features emerge as dominant determinants of LOS in patients with active UC

To identify key factors influencing LOS in patients with active UC, we applied correlation analysis and multivariate negative binomial regression. LOS was positively correlated with mMayo score, IMES, WBC count, PLT count, CRP, and GGT levels. However, LOS was negatively correlated with RBC count, hemoglobin, albumin, TC, and BUN levels (all *p* < 0.05) (Supplementary Table S1). Supplementary Table S2 shows the correlation analysis of clinical features with LOS among UC patients by severity. In the mild subgroup, LOS was positively correlated with ESR and negatively correlated with body mass index, RBC count, hemoglobin, and albumin levels (all *p* < 0.05). In the moderate subgroup, LOS was positively correlated with mMayo score, IMES, WBC count, and CRP level and negatively correlated with RBC count, hemoglobin, and albumin levels (all *p* < 0.05). In the severe subgroup, LOS was positively correlated with CRP level and negatively correlated with RBC count and albumin level (all *p* < 0.05).

The results from multivariate negative binomial regression showed that each-unit increase in mMayo score (one score) and CRP level (mg/L) and the reduction in RBC count (ln 1,012/L) and albumin level (g/L) were associated with an increased LOS and their matched estimated IRRs and 95% CIs were 1.045 (1.023, 1.067), 1.002 (1.000, 1.003), 0.665 (0.525, 0.843), and 0.978 (0.969, 0.987), respectively, among patients with active UC. The results show LOS associations with clinical features stratified by disease severity. Subsequently, we further verified that a reduction in RBC count was associated with increased LOS, and the corresponding estimated IRR and 95% CI for patients in the mild subgroup was 0.322 (0.126, 0.825). In the moderate subgroup, each-unit increase in IMES (one score) and albumin level (g/L) was associated with prolonged LOS, and their corresponding estimated IRRs and 95% CIs were 1.208 (1.024, 1.425) and 0.981 (0.967, 0.995), respectively. In the severe subgroup, each-unit decrease in albumin level (g/L) and RBC count (ln 10^12^/L) was associated with increased LOS, and the corresponding estimated IRR values and 95% CIs were 0.970 (0.948, 0.993) and 0.711 (0.527, 0.958), respectively (Table 5).

**Table 5:** Variables independently associated with length of hospital stay in patients with active UC (*n* = 366).

Variables	Bivariate analysis (IRR, 95% CI)	*p*-value	Multivariable analysis (IRR, 95% CI)	*p*-value
mMayo score	1.081 (1.051, 1.111)	0.000	1.045 (1.023, 1.067)	0.000
IMES	1.385 (1.245, 1.540)	0.000		
WBC	1.023 (1.001, 1.045)	0.045		
RBC	0.494 (0.380, 0.642)	0.000	0.665 (0.525, 0.843)	0.001
HB	0.993 (0.991, 0.996)	0.000		
PLT	1.001 (1.000, 1.001)	0.040		
CRP	1.004 (1.003, 1.006)	0.000	1.002 (1.000, 1.003)	0.002
ALB	0.967 (0.958, 0.975)	0.000	0.978 (0.969, 0.987)	0.000
AST	0.994 (0.986, 1.001)	0.110		
GGT	1.002 (1.000, 1.004)	0.118		
TC	1.002 (1.000, 1.004)	0.055	1.003 (1.001, 1.004)	0.021
BUN	0.995 (0.991, 1.000)	0.052		
Active UC severity				
Mild				
BMI	0.967 (0.907, 1.030)	0.298		
RBC	0.248 (0.097, 0.631)	0.003	0.322 (0.126, 0.825)	0.018
HB	0.991 (0.985, 0.998)	0.007		
ESR	1.006 (1.000, 1.013)	0.050		
ALB	0.978 (0.962, 0.994)	0.007	0.987 (0.971, 1.003)	0.112
Moderate				
mMayo score	1.108 (1.032, 1.190)	0.005		
IMES	1.434 (1.239, 1.659)	0.000	1.208 (1.024, 1.425)	0.003
RBC	0.414 (0.266, 0.646)	0.000	0.683 (0.428, 1.088)	0.108
HB	0.994 (0.991, 0.997)	0.000		
CRP	1.005 (1.003, 1.006)	0.000	1.002 (1.000, 1.004)	0.053
ALB	0.968 (0.958, 0.979)	0.000	0.981 (0.967, 0.995)	0.007
GGT	1.005 (1.002, 1.007)	0.001		
TC	1.002 (1.000, 1.004)	0.044	1.002 (1.001, 1.004)	0.005
Severe				
ALB	0.966 (0.944, 0.989)	0.008	0.970 (0.948, 0.993)	0.011
RBC	0.657 (0.482, 0.894)	0.004	0.711 (0.527, 0.958)	0.025
ALP	0.994 (0.989, 1.000)	0.035		

UC, ulcerative colitis; BMI, body mass index; mMayo, modified Mayo score; IMES, improved Mayo endoscopic score; WBC, white blood cell count; RBC, red blood cell count; HB, hemoglobin; PLT, platelet; CRP, C-reactive protein; ESR, erythrocyte sedimentation rate; ALB, albumin; ALT, alanine aminotransferase; AST, aspartate aminotransferase; ALP, alkaline phosphatase; GGT, gamma-glutamyl transferase; GLU, serum glucose; TC, total cholesterol; BUN, blood urea nitrogen; IRR, incidence rate ratio; 95% CI, 95% confidence interval.

Furthermore, we used correlation analysis and multivariate linear regression to analyze the factors influencing albumin levels in patients with active UC. Supplementary Table S3 showed that albumin was positively correlated with RBC count, hemoglobin, ALT, AST, ALP, and BUN levels, but negatively correlated with WBC and PLT counts (all *p* < 0.05) (Supplementary Table S3). The multivariate linear regression showed that the increases in RBC count, hemoglobin, AST, and ALP levels, as well as the decreases in WBC count and GGT level, were associated with increased albumin, and their corresponding estimated β values and 95% CI were 3.215 (0.671 to 5.759), 10.006 (7.279 to 12.733), 3.680 (2.020 to 5.341), 2.675 (0.566 to 4.785), −2.721 (−4.410 to −1.032), and −1.949 (−2.908 to −0.990), respectively, among patients with active UC (Supplementary Table S4). The data indicated that albumin depletion in active UC, particularly in moderate-to-severe cases, was primarily influenced by reductions in RBC count, hemoglobin level, and transaminase activity.

Hence, the important clinical indicators, including mMayo score, IMES, CRP, RBC count, hemoglobin, and albumin levels, were dominant determinants of LOS in active UC patients, with albumin reduction serving as a particularly prominent marker for extended hospitalization, especially in moderate-to-severe cases.

### 3.5 Albumin level and psychological resilience serve as dominant determinants of LOS in patients with UC in remission stage

Among patients in remission (*n* = 34), LOS was positively correlated with the Comprehensive Score for Financial Toxicity (*r* = 0.397, *p* < 0.05), indicating that higher scores on the scale were associated with lower financial toxicity. However, LOS was negatively correlated with RBC count, albumin level, Connor–Davidson Resilience Scale score, and UWES score (all *p* < 0.05) (Supplementary Table S5). These results suggested that reductions in RBC count and albumin levels, the reduction of financial toxicity, and the depletion of psychological resilience and occupational psychology may be related to an increased LOS. The results from the multivariate negative binomial regression model indicated that the decrease in albumin level and the Connor–Davidson Resilience Scale score were associated with an increased LOS, and their corresponding estimated IRR values and 95% CIs were 0.967 (0.937, 0.997) and 0.434 (0.188, 1.003), respectively (Table 6).

**Table 6:** Association between LOS and selective variables with UC in remission stage (*n* = 34).

Variables	Bivariate analysis (IRR, 95% CI)	*p*-values	Multivariable analysis (IRR, 95% CI)	*p*-values
RBC	0.208 (0.046, 0.937)	0.041		
ALB	0.956 (0.927, 0.986)	0.004	0.967 (0.937, 0.997)	0.016
Comprehensive score for financial toxicity	0.700 (0.473, 1.037)	0.075		
Connor–Davidson resilience scale score	0.336 (0.15, 0.751)	0.008	0.434 (0.188, 1.003)	0.051

UC, ulcerative colitis; RBC, red blood cell count; ALB, albumin; IRR, incidence rate ratio; 95% CI, 95% confidence interval.

Furthermore, among patients in remission, albumin was positively correlated with RBC count and hemoglobin level (all *p* < 0.05) (Supplementary Table S6). The multivariate linear regression showed that an increase in RBC count was associated with increased albumin level, and the corresponding estimated β value and 95% CI were 27.126 (13.133–41.119) (Supplementary Table S7). The data implied that albumin reduction was primarily influenced by the decrease in RBC count among patients in remission.

**Figure 2:**
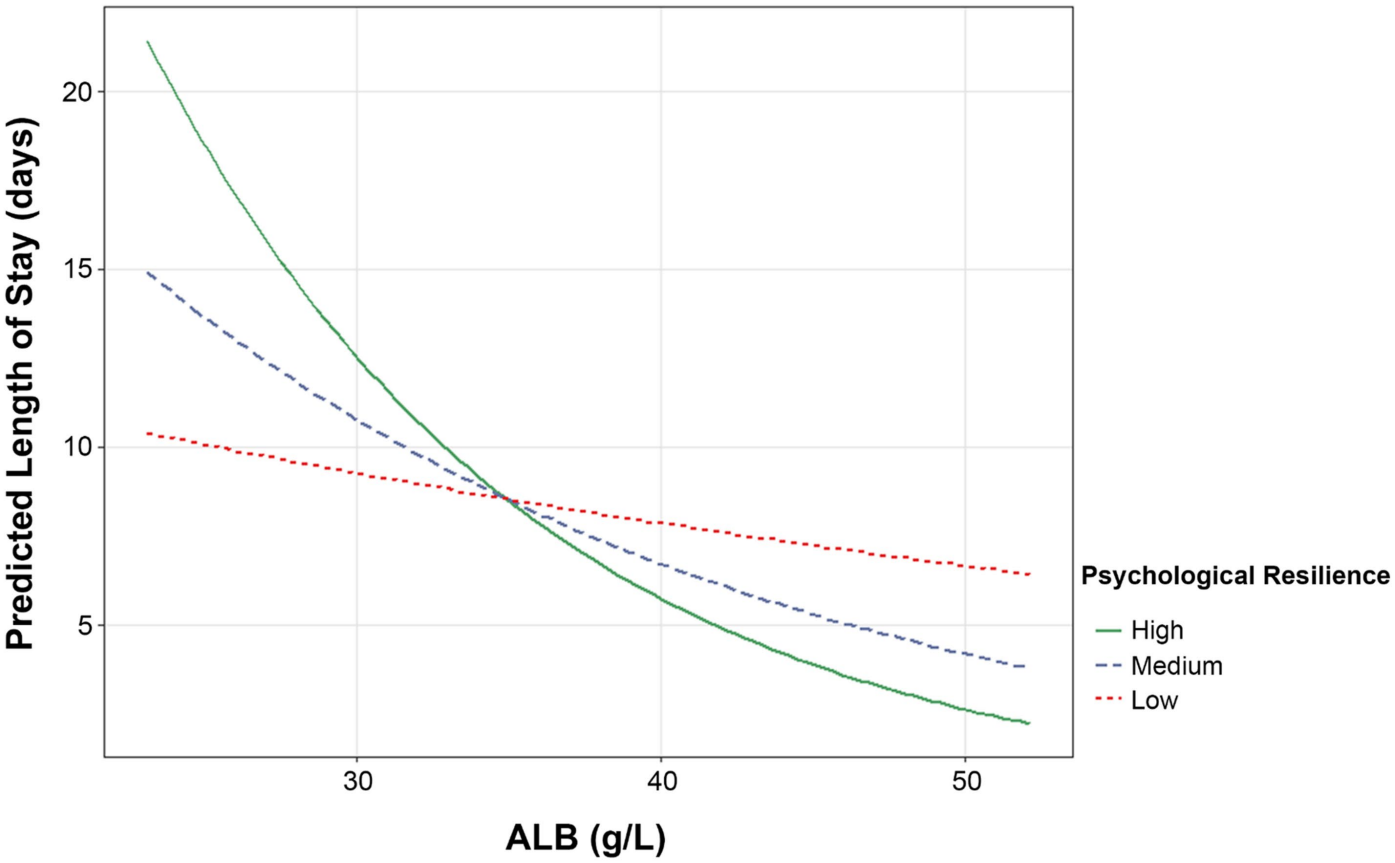
The interaction between the Connor–Davidson Resilience Scale and albumin on LOS in patients with UC in remission was analyzed using linear regression. The X-axis represents albumin levels, and the Y-axis represents the predicted LOS days for low (mean − SD), medium, and high (mean + SD) Connor–Davidson Resilience Scale scores.

These results indicated that albumin served as a potential predictor of LOS for patients in remission, even in the presence of normalized RBC count and hemoglobin levels, hepatic and renal parameters, and significant clinical improvement. This association appears to be related to the nutritional status of patients in remission. Moreover, enhanced psychological resilience may help shorten LOS. Figure 2 shows the interaction of Connor–Davidson Resilience Scale score and albumin on LOS, and the results showed that increased albumin levels were related to a predicted LOS reduction, especially among patients with high Connor–Davidson Resilience Scale scores (P_interaction_ = 0.034).

## 4 Discussion

The principal finding in our research elucidates that clinical features, such as advancement of disease severity and extent, decreases in RBC count and albumin levels, and increases in IMES and CRP levels, emerge as dominant determinants of LOS in patients with active UC. However, both recovered albumin levels and strengthened psychological resilience are beneficial for shortening LOS among patients with UC in the remission stage, implying that targeting clinical inflammation and nutritional status, along with enhancing psychological resilience, can inform stage-specific interventions to reduce hospitalization in UC.

LOS is widely used as a critical indicator of hospital efficiency and healthcare resource utilization (23, 24). A reduction in LOS is often regarded as a powerful proxy metric for improved efficiency in medical resource utilization. In our study, patients with active UC and greater disease extent—left-sided colitis and pancolitis—were also found to have a significantly higher LOS, whereas patients with UC in the remission stage had a lower LOS. These results suggested that patients with severe symptoms and greater disease extent may require more medical resources and impose a greater burden on both family and society. In contrast, those with stable disease have shorter LOS, thereby reducing healthcare expenses and resource use, which highlights the necessity of prompt and effective treatments and health care for patients with active UC to achieve clinical remission. However, an excessively short LOS may reflect inadequate medical resources, economic constraints, or limited bed availability, which could contribute to negative patient experiences and psychological distress (23). Therefore, an appropriate reduction in LOS, which is a major goal for modern healthcare systems, can support efficient patient flow, effective hospital functioning, healthcare cost reduction, and improvement of patients’ psychological wellbeing.

The determinants of LOS in patients with IBD are profoundly dynamic and depend on factors such as disease staging and severity, nutritional status, the presence of IBD-related complications, and the requirement for surgical intervention. Hill et al. (25) found that prolonged postoperative LOS in patients suffering IBD-related surgery was associated with bleeding disorders and hypoalbuminemia. IBD itself is an independent risk factor for increased LOS and operative duration in patients with colectomy (26). One recent study demonstrated that disease severity and the optimal timing of biologic therapy significantly influenced LOS of patients with acute severe UC (27). Consistent with this, our study showed that more advanced disease severity and greater disease extent were positively associated with increased LOS in patients with active UC, indicating that UC progression directly necessitates extended hospitalization to achieve clinical response or remission. Furthermore, we identified several clinical indicators as independent determinants of prolonged LOS in active UC, including elevated mMayo scores, IMES, and CRP levels, as well as reduced RBC count and albumin levels. Additionally, albumin level emerged as a dominant determinant of LOS in patients with UC both in the active and remission stages. This underscores that improving clinical symptoms may effectively shorten LOS in patients with active UC. It further emphasized that nutritional recovery, particularly in albumin level, played a significant role in shortening LOS regardless of disease phase.

Low serum albumin level is closely associated with adverse outcomes and prolonged LOS in multiple diseases (28, 29), such as acute coronary syndrome (30), acute heart failure (31), and respiratory failure (32). Pan et al. (30) found that poor albumin status was a critical risk factor for prolonged LOS in patients undergoing percutaneous coronary intervention for acute coronary syndrome, while improving albumin levels could reduce LOS. Albumin levels are predictive of disease severity and therapeutic efficacy in IBD (33, 34). Similarly, we found low albumin levels correlated with worsening disease severity in UC. Furthermore, our data showed that low albumin level was a primary factor influencing prolonged LOS in patients with UC during both active and remission stages. Anemia and hypoalbuminemia are common features in patients with IBD, which are strongly associated with disease severity (35). Additionally, the co-variance in albumin levels and transaminase activity in IBD reflects underlying metabolic dysregulation and immune dysfunction (36). We found that in patients with active UC who had normal hepatic and renal function, low albumin levels were associated with reduced RBC count and hemoglobin levels; moreover, a positive correlation was observed between albumin levels and transaminase activity. These findings indicate that albumin depletion in active UC occurs through both nutritional losses induced by intestinal bleeding and metabolic consumption driven by systemic inflammation and immune dysfunction. In addition, the absence of intestinal bleeding and a decrease in stool frequency are characteristics of UC in clinical remission (11). However, we found that albumin levels in patients with UC in clinical remission might still be linked to RBC count, suggesting that the association points to potentially nutritional status rather than active inflammation. Hence, improving albumin levels by controlling intestinal bleeding and reducing stool frequency in active UC and increasing nutritional intake in remission contribute to shortening LOS and improving prognosis.

Numerous studies have shown that psychological factors emerge as important determinants of LOS in patients with various diseases. Burn patients with a psychiatric history experience prolonged LOS due to the challenges of physical recovery and psychosocial management (37, 38). Velelekou et al. (39) found that, beyond clinical factors, basic psychosocial factors were remarkably associated with LOS in patients with schizophrenia. In our study, LOS in patients with UC in remission stage was negatively correlated with Connor–Davidson Resilience Scale and UWES scores, indicating that strengthening psychological resilience and occupational psychology could contribute to shortened LOS and sustained clinical remission of UC. Thus, both the recovery of nutritional parameters such as albumin levels and the fortification of psychological resilience help shorten LOS in patients with UC in remission, thereby reducing healthcare expenditures, facilitating the optimal use of medical resources, and maximizing bed occupancy.

In patients with IBD, chronic colon inflammation exacerbates psychological stress, and this stress subsequently aggravates UC progression through perturbing intestinal microbiome homeostasis and inducing immune dysfunction, thereby exacerbating IBD progression (40–42). However, during periods of heightened disease activity, some patients manifest strengthened psychological resilience to cope with the stresses of a chronic illness (43). Our analysis demonstrated a significantly higher median Connor–Davidson Resilience Scale score in patients with active UC than in those with UC in remission. Moreover, it seems that higher disease severity is associated with a higher median Connor–Davidson Resilience Scale score in patients with active UC. These findings suggest that confronting UC progression may enhance psychological resilience in patients. Additionally, psychological assets may act as buffers, facilitating an efficient recovery process (44). Therefore, strengthening psychological resilience is a crucial adjunctive strategy, contributing to the alleviation of clinical symptoms and the attainment of remission.

Financial toxicity is used to describe the multifaceted financial burden associated with medical treatment, which triggers psychological distress for patients. Financial toxicity among patients with IBD is pervasive, owing to the nature of IBD being prone to deterioration and relapse, recurrent hospitalizations, and the high risk of colorectal cancer and surgery (45–47). In the United States, patients with severe and active IBD may experience high financial distress due to high medical costs. This distress is multifactorial, linked to a range of clinical and socioeconomic factors, including severity of IBD, comorbid illnesses, lower education level, lower household income, and public insurance (47, 48). Uninsured patients undergoing IBD-related surgery are particularly vulnerable to financial toxicity, whereas those with insurance face considerably lower risks (45). Similarly, in Spain, IBD patients endure significant disease and financial burdens, further straining the healthcare system (49). In China, patients with IBD suffering opportunistic infections could result in hospital stays and medical costs (50). However, our study revealed no significant difference in the median Comprehensive Score for Financial Toxicity between patients with UC in active and remission stages. Moreover, 98% of patients were covered by China’s medical insurance system, indicating that, despite the expected higher medical expenses during active UC, financial toxicity is nearly absent in China. These findings suggest that the near-universal coverage of the national medical insurance scheme serves as a critical buffer, protecting against the financial distress exacerbated by unpredictable disease courses.

In addition, robust family and social support are critical components of comprehensive IBD care, providing benefits to patients. Family participatory nursing is beneficial for improving patients’ clinical outcomes, shortening LOS, and enhancing families’ psychological status, thereby reducing the severity of flare-ups and improving the overall management of acute-phase disease (51). Estée C H Feldman et al. revealed that younger and middle-aged adolescents with IBD derived remarkable benefits from family-supported disease management (52). In addition, the lack of social support could potentiate financial toxicity in patients with IBD (46). Enhancing social support and psychological adaptability can improve the management of patients with IBD and overall wellbeing (53). Consequently, proactively addressing biopsychosocial factors presents an important strategy for shortening LOS, improving patients’ outcomes, delivering personalized medicine, and boosting overall healthcare efficiency.

There are some limitations when interpreting the findings. First, because this study is a retrospective study design, causal associations cannot be established, as the observed associations between LOS and its determinants cannot be interpreted as cause-and-effect relationships. Therefore, a larger and multicenter cohort is required to verify the role of biopsychosocial factors in LOS among patients with UC, and future research should focus on interventions, particularly randomized controlled trials testing the efficacy of psychological resilience–building programs on clinical outcomes such as LOS and readmission rates in this population. Second, the generalizability of our findings is constrained by the single-center setting and local clinical protocols; therefore, the results must be extrapolated to other populations and healthcare contexts with caution. Third, the questionnaire was used to collect self-reported information, introducing the potential for recall bias. However, the questionnaire demonstrated moderate-to-high internal consistency in this study. Fourth, given our observation that intestinal bleeding–induced anemia may reduce albumin levels in active UC, a future study will quantify the extent of this relationship. Finally, unmeasured factors such as genetic and environmental factors may have confounded the findings of this study.

## 5 Conclusion

In summary, the study highlights the evolving understanding of LOS determinants in UC. Biomedical factors remain the dominant drivers of LOS in patients with active UC, particularly through improvements in nutritional parameters, such as albumin levels. Furthermore, beyond physiological restoration, enhanced psychological resilience has been identified as an increasingly important factor associated with improved LOS outcomes in patients in remission. This study’s findings suggest that biomarker-guided nutritional support, particularly albumin optimization, may serve as an effective strategy for reducing LOS in active UC, and integrating psychological resilience–building interventions into standard care could further shorten hospitalization and improve outcomes for patients in remission, which may benefit not only UC patients and their families but also the clinical staff specializing in gastroenterology, and health administrators.

## Data Availability

The raw data supporting the conclusions of this article will be made available by the authors, without undue reservation.
