# Supplementary material for: Albumin and psychological resilience: key modifiable factors for hospital length of stay in ulcerative colitis
Source: Front Nutr. 2026 Jan 8;12:1704658. doi: 10.3389/fnut.2025.1704658 (PMC12823499; doi:10.3389/fnut.2025.1704658)
Supplement: Supplementary file 1 [file Table_1.docx]

**Table S1 The correlation between length of stay with clinical indexes and scales in patients with active UC (n = 366)**

| **Indexes** | **Length of stay (LOS)** | |
| --- | --- | --- |
|  | ***r*** | **P-value** |
| Age | 0.058 | 0.268 |
| Duration of UC | -0.021 | 0.686 |
| mMayo score | **0.286** | **0.000^a^** |
| IMES | **0.276** | **0.000^a^** |
| BMI | 0.010 | 0.851^a^ |
| **Complete Blood Count and inflammation indexes** |  |  |
| WBC | **0.173** | **0.001^a^** |
| RBC | **-0.279** | **0.000^a^** |
| HB | **-0.255** | **0.000^a^** |
| PLT | **0.140** | **0.007^a^** |
| CRP | **0.351** | **0.000^a^** |
| ESR | 0.115 | 0.053^a^ |
| **Hepatic parameters** |  |  |
| ALB | **-0.412** | **0.000^a^** |
| ALT | -0.048 | 0.361^a^ |
| AST | **-0.136** | **0.009^a^** |
| AST/ALT Ratio | -0.026 | 0.621^a^ |
| ALP | -0.081 | 0.125^a^ |
| GGT | **0.147** | **0.005^a^** |
| **Blood Glucose and Lipid parameters** |  |  |
| GLU | -0.031 | 0.561^a^ |
| TC | **-0.204** | **0.000^a^** |
| TG | -0.029 | 0.599^a^ |
| **Renal parameters** |  |  |
| BUN | **-0.137** | **0.009^a^** |
| SCR | -0.044 | 0.409^a^ |
| **Assessment of Scales** |  |  |
| Comprehensive Score for Financial Toxicity | 0.042 | 0.428^a^ |
| PHQ-9 score | 0.008 | 0.878^a^ |
| Family Communication Scale score | -0.002 | 0.963^a^ |
| Connor-Davidson Resilience Scale score | -0.031 | 0.552^a^ |
| Self-Management Behavior Scale score | 0.012 | 0.823^a^ |
| Social Support Scale score | 0.030 | 0.568^a^ |
| Utrecht Work Engagement Scale score | -0.068 | 0.195^a^ |

^a^ Spearmen's correlation was used to analysis of each non-normally distributed variables.

**Abbreviations:** SD: Standard deviation; UC: Ulcerative Colitis; BMI: Body Mass Index; mMayo: modified Mayo; IMES: Improved Mayo Endoscopic Score; WBC: White blood cell count; RBC: Red blood cell count; HB: Hemoglobin; PLT: Platelet; CRP: C-reactive protein; ESR: Erythrocyte sedimentation rate; ALB: Albumin; ALT: Alanine aminotransferase; AST: Aspartate aminotransferase; ALP: Serum alkaline phosphatase; GGT: Gamma-Glutamyl transferase; GLU: Serum glucose; TC: Total cholesterol; TG: Triglyceride; BUN: Blood urea nitrogen; SCR: Serum creatinine; PHQ-9: Patient Health Questionnaire-9.

**Table S2 The correlation between length of stay with clinical indexes and scales in patients with active UC** **in three subgroups (the Mild, Moderate, and Severe groups)**

| **Indexes** | **Mild (n = 58)** | |  | **Moderate (n = 231)** | |  | **Severe (n = 77)** | |
| --- | --- | --- | --- | --- | --- | --- | --- | --- |
|  | ***r*** | **P-value** |  | ***r*** | **P-value** |  | ***r*** | **P-value** |
| Age | 0.024 | 0.859^a^ |  | 0.067 | 0.309^a^ |  | 0.054 | 0.643^a^ |
| Duration of UC | 0.106 | 0.429^a^ |  | -0.039 | 0.557^a^ |  | -0.117 | 0.312^a^ |
| mMayo score | -0.140 | 0.295^a^ |  | **0.221** | **0.001^a^** |  | 0.122 | 0.290^a^ |
| IMES | -0.116 | 0.385^a^ |  | **0.275** | **0.000^a^** |  | -0.002 | 0.984^a^ |
| BMI | **-0.265** | **0.045^a^** |  | 0.059 | 0.371^a^ |  | 0.125 | 0.279^a^ |
| **Complete Blood Count and inflammation indexes** |  |  |  |  |  |  |  |  |
| WBC | 0.172 | 0.202^a^ |  | **0.188** | **0.004^a^** |  | 0.051 | 0.661^a^ |
| RBC | **-0.272** | **0.040^a^** |  | **-0.201** | **0.002^a^** |  | **-0.383** | **0.001^a^** |
| HB | **-0.276** | **0.038^a^** |  | **-0.219** | **0.001^a^** |  | -0.219 | 0.056^a^ |
| PLT | 0.187 | 0.163^a^ |  | 0.114 | 0.084^a^ |  | 0.026 | 0.825^a^ |
| CRP | 0.164 | 0.219^a^ |  | **0.351** | **0.000^a^** |  | **0.289** | **0.011^a^** |
| ESR | **0.377** | **0.016^a^** |  | 0.131 | 0.080^a^ |  | -0.197 | 0.116^a^ |
| **Hepatic parameters** |  |  |  |  |  |  |  |  |
| ALB | **-0.411** | **0.001^a^** |  | **-0.385** | **0.000^a^** |  | **-0.348** | **0.002^a^** |
| ALT | -0.202 | 0.133^a^ |  | -0.009 | 0.892^a^ |  | -0.015 | 0.898^a^ |
| AST | -0.017 | 0.899^a^ |  | **-0.136** | **0.039^a^** |  | 0.000 | 0.997^a^ |
| AST/ALT Ratio | 0.259 | 0.051^a^ |  | -0.085 | 0.199^a^ |  | 0.034 | 0.770^a^ |
| ALP | 0.198 | 0.140^a^ |  | -0.046 | 0.487^a^ |  | **-0.287** | **0.012^a^** |
| GGT | -0.069 | 0.608^a^ |  | **0.229** | **0.000^a^** |  | -0.026 | 0.822^a^ |
| **Blood Glucose and Lipid parameters** |  |  |  |  |  |  |  |  |
| GLU | -0.032 | 0.818^a^ |  | -0.035 | 0.608^a^ |  | -0.017 | 0.889^a^ |
| TC | -0.174 | 0.232^a^ |  | **-0.185** | **0.008^a^** |  | -0.152 | 0.210^a^ |
| TG | 0.092 | 0.531^a^ |  | 0.032 | 0.645^a^ |  | **-0.269** | **0.024^a^** |
| **Renal parameters** |  |  |  |  |  |  |  |  |
| BUN | -0.044 | 0.746^a^ |  | -0.092 | 0.165^a^ |  | -0.227 | 0.050^a^ |
| SCR | -0.172 | 0.200^a^ |  | -0.012 | 0.860^a^ |  | 0.004 | 0.971^a^ |
| **Assessment of Scales** |  |  |  |  |  |  |  |  |
| Comprehensive Score for Financial Toxicity | -0.056 | 0.675^a^ |  | 0.019 | 0.777^a^ |  | 0.197 | 0.086^a^ |
| PHQ-9 score | 0.191 | 0.152^a^ |  | -0.044 | 0.510^a^ |  | -0.054 | 0.641^a^ |
| Family Communication Scale score | -0.107 | 0.426^a^ |  | 0.012 | 0.859^a^ |  | 0.133 | 0.247^a^ |
| Connor-Davidson Resilience Scale score | 0.195 | 0.142^a^ |  | -0.071 | 0.284^a^ |  | -0.102 | 0.375^a^ |
| Self-Management Behavior Scale score | 0.123 | 0.359^a^ |  | -0.030 | 0.647^a^ |  | -0.018 | 0.876^a^ |
| Social Support Scale score | 0.055 | 0.680^a^ |  | 0.024 | 0.711^a^ |  | 0.069 | 0.554^a^ |
| Utrecht Work Engagement Scale score | -0.051 | 0.702^a^ |  | -0.036 | 0.584^a^ |  | -0.078 | 0.500^a^ |

^a^ Spearmen's correlation was used to analysis of each non-normally distributed variables.

**Abbreviations:** SD: Standard deviation; UC: Ulcerative Colitis; BMI: Body Mass Index; mMayo: modified Mayo; IMES: Improved Mayo Endoscopic Score; WBC: White blood cell count; RBC: Red blood cell count; HB: Hemoglobin; PLT: Platelet; CRP: C-reactive protein; ESR: Erythrocyte sedimentation rate; ALB: Albumin; ALT: Alanine aminotransferase; AST: Aspartate aminotransferase; ALP: Serum alkaline phosphatase; GGT: Gamma-Glutamyl transferase; GLU: Serum glucose; TC: Total cholesterol; TG: Triglyceride; BUN: Blood urea nitrogen; SCR: Serum creatinine; PHQ-9: Patient Health Questionnaire-9.

**Table S3 The correlation between** **Albumin with Complete Blood Count and other Hepato-Renal parameters in patients with active UC (n = 366)**

| **Indexes** | **ALB** | |
| --- | --- | --- |
|  | ***r*** | **P-value** |
| Age | -0.083 | 0.113^b^ |
| BMI | 0.000 | 0.993^a^ |
| **Complete Blood Count** |  |  |
| WBC | **-0.193** | **0.000^b^** |
| RBC | **0.480** | **0.000^b^** |
| HB | **0.519** | **0.000^b^** |
| PLT | **-0.270** | **0.000^b^** |
| **Hepatic parameters** |  |  |
| ALT | **0.250** | **0.000^b^** |
| AST | **0.374** | **0.000^b^** |
| AST/ALT Ratio | -0.043 | 0.417^b^ |
| ALP | **0.189** | **0.000^b^** |
| GGT | -0.077 | 0.143^b^ |
| **Renal parameters** |  |  |
| BUN | **0.255** | **0.000^b^** |
| SCR | 0.098 | 0.062^a^ |

^a^ Pearson's correlation was used to analysis of each continuous and approximately normally distributed variables; ^b^ Spearmen's correlation was used to analysis of each non-normally distributed variables.

**Abbreviations:** UC: Ulcerative Colitis; ALB: Albumin; BMI: Body Mass Index; WBC: White blood cell count; RBC: Red blood cell count; HB: Hemoglobin; PLT: Platelet; ALT: Alanine aminotransferase; AST: Aspartate aminotransferase; ALP: Serum alkaline phosphatase; GGT: Gamma-Glutamyl transferase; BUN: Blood urea nitrogen; SCR: Serum creatinine.

**Table S4 The association between Albumin with Complete Blood Count and other Hepato-Renal parameters in patients with UC (n = 366)**

| **Variables** | **β value** | **95% confidence interval (CI)** | **P-value** |
| --- | --- | --- | --- |
| WBC (Ln) | -2.721 | -4.410 – -1.032 | 0.002 |
| RBC (Ln) | 3.215 | 0.671 – 5.759 | 0.013 |
| HB (Ln) | 10.006 | 7.279 – 12.733 | 0.000 |
| AST (Ln) | 3.680 | 2.020 – 5.341 | 0.000 |
| ALP (Ln) | 2.675 | 0.566 – 4.785 | 0.013 |
| GGT (Ln) | -1.949 | -2.908 – -0.990 | 0.000 |
| **Active UC severity** |  |  |  |
| **Mild** |  |  |  |
| ALT (Ln) | 5.689 | 1.870 – 9.508 | 0.004 |
| **Moderate** |  |  |  |
| WBC (Ln) | -2.215 | -4.167 – -0.264 | 0.026 |
| RBC (Ln) | 9.690 | 3.881 – 15.500 | 0.001 |
| HB (Ln) | 8.085 | 3.984 – 12.187 | 0.000 |
| AST (Ln) | 3.933 | 2.034 – 5.832 | 0.000 |
| ALP (Ln) | 3.295 | 0.908 – 5.683 | 0.007 |
| GGT (Ln) | -2.584 | -3.638 – -1.531 | 0.000 |
| **Severe** |  |  |  |
| HB (Ln) | 8.149 | 3.830 – 12.468 | 0.000 |

**Abbreviations:** UC: Ulcerative Colitis; WBC: White blood cell count; RBC: Red blood cell count; HB: Hemoglobin; AST: Aspartate aminotransferase; ALP: Serum alkaline phosphatase; GGT: Gamma-Glutamyl transferase.

**Table S5 The correlation between length of stay with clinical indexes and scales in patients with UC in clinical remission (n = 34)**

| **Indexes** | **Length of stay (LOS)** | |
| --- | --- | --- |
|  | ***r*** | **P-value** |
| Age | 0.240 | 0.172^a^ |
| Duration of UC | -0.161 | 0.364^a^ |
| mMayo score | -0.270 | 0.122^a^ |
| IMES | -0.295 | 0.090^a^ |
| BMI | 0.253 | 0.149^a^ |
| **Complete Blood Count and inflammation indexes** |  |  |
| WBC | 0.262 | 0.141^a^ |
| RBC | **-0.386** | **0.026^a^** |
| HB | -0.301 | 0.089^a^ |
| PLT | -0.006 | 0.973^a^ |
| CRP | -0.109 | 0.541^a^ |
| ESR | 0.060 | 0.812^a^ |
| **Hepatic parameters** |  |  |
| ALB | **-0.488** | **0.003^a^** |
| ALT | -0.185 | 0.294^a^ |
| AST | -0.234 | 0.184^a^ |
| AST/ALT Ratio | 0.108 | 0.542^a^ |
| ALP | 0.078 | 0.660^a^ |
| GGT | 0.275 | 0.116^a^ |
| **Blood Glucose and Lipid parameters** |  |  |
| GLU | -0.151 | 0.393^a^ |
| TC | -0.196 | 0.299^a^ |
| TG | -0.089 | 0.640^a^ |
| **Renal parameters** |  |  |
| BUN | -0.036 | 0.840^a^ |
| SCR | 0.181 | 0.306^a^ |
| **Assessment of Scales** |  |  |
| Comprehensive Score for Financial Toxicity | **0.397** | **0.020^a^** |
| PHQ-9 score | 0.245 | 0.162^a^ |
| Family Communication Scale score | -0.026 | 0.883^a^ |
| Connor-Davidson Resilience Scale score | **-0.464** | **0.006^a^** |
| Self-Management Aehavior Scale score | 0.112 | 0.528^a^ |
| Social Support Scale score | 0.031 | 0.863^a^ |
| Utrecht Work Engagement Scale score | **-0.491** | **0.003^a^** |

^a^ Spearmen's correlation was used to analysis of each non-normally distributed variables.

**Aaareviations:** SD: Standard deviation; UC: Ulcerative Colitis; mMayo: modified Mayo; IMES: Improved Mayo Endoscopic Score; BMI: Body Mass Index; WBC: White blood cell count; RBC: Red blood cell count; HB: Hemoglobin; PLT: Platelet; CRP: C-reactive protein; ESR: Erythrocyte sedimentation rate; ALB: Albumin; ALT: Alanine aminotransferase; AST: Aspartate aminotransferase; ALP: Serum alkaline phosphatase; GGT: Gamma-Glutamyl transferase; GLU: Serum glucose; TC: Total cholesterol; TG: Triglyceride; BUN: Blood urea nitrogen; SCR: Serum creatinine; PHQ-9: Patient Health Questionnaire-9.

**Table S6 The correlation between Albumin with Complete Blood Count and other Hepato-Renal parameters in patients with UC in clinical remission (n = 34)**

| **Indexes** | **ALB** | |
| --- | --- | --- |
|  | ***r*** | **P-value** |
| Age | -0.130 | 0.463^b^ |
| BMI | -0.044 | 0.806^a^ |
| **Complete Blood Count** |  |  |
| WBC | 0.023 | 0.897^b^ |
| RBC | **0.573** | **0.000^b^** |
| HB | **0.527** | **0.002^b^** |
| PLT | 0.028 | 0.876^b^ |
| **Hepatic parameters** |  |  |
| ALT | 0.287 | 0.100^b^ |
| AST | 0.240 | 0.172^b^ |
| AST/ALT Ratio | -0.121 | 0.494^b^ |
| ALP | -0.174 | 0.326^b^ |
| GGT | 0.159 | 0.368^b^ |
| **Renal parameters** |  |  |
| BUN | 0.019 | 0.913^b^ |
| SCR | 0.046 | 0.797^a^ |

^a^ Pearson's correlation was used to analysis of each continuous and approximately normally distributed variables; ^b^ Spearmen's correlation was used to analysis of each non-normally distributed variables.

**Abbreviations:** UC: Ulcerative Colitis; ALB: Albumin; BMI: Body Mass Index; WBC: White blood cell count; RBC: Red blood cell count; HB: Hemoglobin; PLT: Platelet; ALT: Alanine aminotransferase; AST: Aspartate aminotransferase; ALP: Serum alkaline phosphatase; GGT: Gamma-Glutamyl transferase; BUN: Blood urea nitrogen; SCR: Serum creatinine.

**Table S7 The association between Albumin with Complete Blood Count and other Hepato-Renal parameters in patients with UC in clinical remission (n = 34)**

| **Variables** | **β value** | **95% confidence interval (CI)** | **P-value** |
| --- | --- | --- | --- |
| RBC (Ln) | 27.126 | 13.133 – 41.119 | 0.000 |

**Abbreviations:** UC: Ulcerative Colitis; WBC: White blood cell count; RBC: Red blood cell count; HB: Hemoglobin; AST: Aspartate aminotransferase; ALP: Serum alkaline phosphatase; GGT: Gamma-Glutamyl transferase.

**Questionnaire of Patients with UC**

***Demographic and Socioeconomic Information***

**Age**: ____ years

**Gender**: □ Male □ Female

**Ethnicity**: □ Han □ Hui □ Zhuang □ Other

**Marital Status**: □ Single □ Married □ Separated □ Divorced □ Widowed

**Occupation**:

□ Government Official □ Enterprise Staff □ Company Employee □ Self-employed

□ Farmer □ Freelancer □ Educator □ Healthcare Worker

□ Retiree □ Laborer □ Other: ______

**Time Since Diagnosis**: □ <1 year □ 1–5 years □ 5–10 years □ >10 years

**Place of Residence**: □ Rural □ Township □ Urban

**Type of Health Insurance**: □ Provincial/Municipal Insurance □ Resident Insurance □ Commercial Insurance □ Employee Insurance □ New Rural Cooperative Medical System □ None

Primary Caregiver: □ Spouse □ Children □ Nanny □ Friend □ Other: ______

***Socioeconomic Status***

**Education Level**: □ Primary School or Below □ Junior High/Vocational School □ Senior High School □ College or Above

**Occupation**: □ Unemployed □ Migrant Worker □ Retired □ Company Staff/Teacher □ Professor/Manager

**Average Monthly Household Income**: □ <3,000 RMB □ 3,000–5,000 RMB □ 5,000–10,000 RMB □ >10,000 RMB

**Reimbursement Rate of Medical Expenses**: □ Fully Self-funded □ <25% □ 25%–<50% □ 50%–<75% □ >75%

**Household Structure**

□ Nuclear Family (Parents and Unmarried Children)

□ Stem Family (Parents and Children)

□ Couple Only

□ Skipped-generation Family (Grandparents and Grandchildren)

□ Single-parent Family

□ Other: ______

1. ***Comprehensive Score for Financial Toxicity***

Please evaluate each item based on how well it describes your condition over the past 7 days.

Score: 0 = Not at all true, 4 = Completely true

| **Item** | **0** | **1** | **2** | **3** | **4** |
| --- | --- | --- | --- | --- | --- |
| 1. I am confident I have enough savings, pensions, or assets to afford my treatment. |  |  |  |  |  |
| 2. Out-of-pocket costs far exceeded my expectations. |  |  |  |  |  |
| 3. I am very worried that my illness/treatment will lead to serious financial problems. |  |  |  |  |  |
| 4. I feel I have no choice in managing the overall treatment costs. |  |  |  |  |  |
| 5. I am frustrated by my reduced capacity to work or contribute as before. |  |  |  |  |  |
| 6. I am satisfied with my current financial status. |  |  |  |  |  |
| 7. I am able to meet my monthly living expenses. |  |  |  |  |  |
| 8. I feel financially stressed. |  |  |  |  |  |
| 9. I worry about maintaining my current job and income. |  |  |  |  |  |
| 10. My illness/treatment has made me dissatisfied with my financial situation. |  |  |  |  |  |
| 11. I feel I am in control of my financial condition. |  |  |  |  |  |

1. ***PHQ-9: Depression Screening (Past 2 Weeks)***

| **Item** | **0 (Not at all)** | **1 (Several days)** | **2 (More than 7 days)** | **3 (Nearly every day)** |
| --- | --- | --- | --- | --- |
| 1. Little interest or pleasure in doing things |  |  |  |  |
| 2. Feeling down, depressed, or hopeless |  |  |  |  |
| 3. Trouble falling/staying asleep, or sleeping too much |  |  |  |  |
| 4. Feeling tired or having little energy |  |  |  |  |
| 5. Poor appetite or overeating |  |  |  |  |
| 6. Feeling bad about yourself or that you are a failure |  |  |  |  |
| 7. Trouble concentrating, e.g., reading newspaper or watching TV |  |  |  |  |
| 8. Moving/speaking slowly or being unusually restless |  |  |  |  |
| 9. Thoughts of being better off dead or self-harm |  |  |  |  |

### *****Family Communication Scale*****

Rate how accurately each item describes communication in your family:

| **Item** | **1 (Very True)** | **2 (True)** | **3 (Not True)** | **4 (Completely False)** |
| --- | --- | --- | --- | --- |
| 1. Family members understand each other's worries. |  |  |  |  |
| 2. You can’t understand someone’s thoughts from conversation. |  |  |  |  |
| 3. We openly discuss matters instead of hinting. |  |  |  |  |
| 4. We rarely express affection verbally. |  |  |  |  |
| 5. We speak straightforwardly rather than indirectly. |  |  |  |  |
| 6. We often hide our thoughts. |  |  |  |  |
| 7. We are frank with each other. |  |  |  |  |
| 8. We stop talking when angry. |  |  |  |  |
| 9. We point out things we dislike about each other. |  |  |  |  |

1. ***Psychological Resilience Scale***

Instructions: The following self-assessment scale is used to evaluate psychological resilience. Please select the option that best describes your condition over the past month. There are no right or wrong answers.

| Item | Completely Untrue (0) | Mostly Untrue (1) | Neutral (2) | Mostly True (3) | Completely True (4) |
| --- | --- | --- | --- | --- | --- |
| 1. I can adapt flexibly to changes. |  |  |  |  |  |
| 2. I can cope with difficulties when they arise. |  |  |  |  |  |
| 3. I respond to problems with humor. |  |  |  |  |  |
| 4. I have become stronger through accumulated experiences. |  |  |  |  |  |
| 5. I am highly resilient after illness or hardship. |  |  |  |  |  |
| 6. I can achieve my goals even when encountering obstacles. |  |  |  |  |  |
| 7. I can concentrate under pressure. |  |  |  |  |  |
| 8. I do not get discouraged by failure. |  |  |  |  |  |
| 9. I believe I am a strong person when facing life's challenges. |  |  |  |  |  |
| 10. I am capable of managing unpleasant emotions, such as anger. |  |  |  |  |  |

### *****Self-Management Behavior Scale*****

This questionnaire investigates your self-management behaviors related to IBD. Please mark "√" in the box that best represents your actual situation.

| **Item** | **Never (1)** | **Rarely (2)** | **Sometimes (3)** | **Often (4)** | **Always (5)** |
| --- | --- | --- | --- | --- | --- |
| 1. Are you familiar with the usage, dosage, effects, side effects, and precautions of your IBD medications? |  |  |  |  |  |
| 2. Can you follow healthcare providers' instructions in using IBD medications (on time, correct dose, complete course)? |  |  |  |  |  |
| 3. Can you continue taking your medication as required when symptoms are well controlled? |  |  |  |  |  |
| 4. Are you able to detect adverse reactions to IBD medications in time? |  |  |  |  |  |
| 5. Do you communicate promptly with your doctor when adverse effects or disease progression occurs, and adjust medication accordingly? |  |  |  |  |  |
| 6. Can you control your intake of sweets (e.g., chocolate, gum, cola), meat, and high-fat foods? |  |  |  |  |  |
| 7. Can you limit intake of processed snacks with additives or preservatives? |  |  |  |  |  |
| 8. Can you restrict fast food or takeaway consumption? |  |  |  |  |  |
| 9. Do you pay attention to healthy cooking methods (e.g., boiling/steaming instead of frying)? |  |  |  |  |  |
| 10. Do you regularly monitor your weight to manage nutrition? |  |  |  |  |  |
| 11. Can you control consumption of raw, cold, hard, spicy, greasy, fibrous foods (e.g., corn, bamboo shoots, leeks, celery, cauliflower), seafood, etc.? |  |  |  |  |  |
| 12. Do you ensure a nutritionally balanced diet? |  |  |  |  |  |
| 13. Do you keep a food diary to identify intolerances? |  |  |  |  |  |
| 14. Do you avoid binge eating? |  |  |  |  |  |
| 15. Do you closely monitor your bowel movements (frequency and appearance)? |  |  |  |  |  |
| 16. Do you attend regular follow-up visits as advised by your doctor? |  |  |  |  |  |
| 17. Do you monitor your abdominal symptoms (e.g., pain, lumps)? |  |  |  |  |  |
| 18. Are you able to recognize signs of disease relapse promptly? |  |  |  |  |  |
| 19. When feeling low, can you encourage yourself to recover emotionally? |  |  |  |  |  |
| 20. When feeling low, do you seek emotional relief (e.g., walking, music, movies)? |  |  |  |  |  |
| 21. Do you make efforts to cultivate positive emotions to aid recovery? |  |  |  |  |  |
| 22. When you feel "useless", do you try to change this mindset? |  |  |  |  |  |
| 23. When you feel like a burden to your family, do you try to overcome that thought? |  |  |  |  |  |
| 24. When complications occur, can you remain emotionally stable? |  |  |  |  |  |
| 25. Can you choose appropriate physical activity (type, intensity, duration) based on your condition or professional advice? |  |  |  |  |  |
| 26. Can you adjust your exercise routines according to disease changes? |  |  |  |  |  |
| 27. Do you rehydrate and replenish electrolytes appropriately after exercise? |  |  |  |  |  |
| 28. Do you maintain a regular schedule (early to bed and rise, sufficient sleep)? |  |  |  |  |  |
| 29. Can you avoid overexertion? |  |  |  |  |  |
| 30. Can you balance work, activities, and rest? |  |  |  |  |  |
| 31. Do you participate in social activities and maintain healthy relationships? |  |  |  |  |  |
| 32. When feeling down, do you confide in family or friends for comfort or help? |  |  |  |  |  |

### *Social Support Rating Scale*

1.How many close friends do you have who can provide support and assistance?
□ None □ 1–2 □ 3–5 □ 6 or more

2.In the past year, your living situation was:
□ Lived alone, isolated from family □ Frequently changed residences, mostly with strangers
□ Lived with classmates, coworkers, or friends □ Lived with family

3.Relationship with neighbors:
□ Indifferent, nodding acquaintances □ Slight concern when difficulties arise
□ Somewhat caring □ Most are very caring

4.Relationship with colleagues:
□ Indifferent □ Slight concern in difficulty □ Most are very caring

5.Support and care from family members:

| **Family Member** | **None** | **Very Little** | **Some** | **Full Support** |
| --- | --- | --- | --- | --- |
| Spouse |  |  |  |  |
| Parents |  |  |  |  |
| Children |  |  |  |  |
| Siblings |  |  |  |  |
| Others (e.g., in-laws) |  |  |  |  |

6.Sources of financial or practical help during emergencies:
□ No sources □ The following sources (check all that apply):
A. Spouse B. Other family members C. Friends D. Relatives E. Colleagues F. Workplace
G. Official/semi-official organizations (e.g., unions) H. Religious/social non-governmental organizations

7.Sources of emotional comfort during emergencies:
□ No sources □ The following sources (check all that apply):
A. Spouse B. Other family members C. Friends D. Relatives E. Colleagues F. Workplace
G. Official/semi-official organizations H. Religious/social non-governmental organizations I. Others (specify)

8.When distressed, how do you express yourself?
□ Never tell anyone □ Only tell 1–2 closest people □ Tell if asked □ Actively share to seek understanding

9.When in trouble, how do you seek help?
□ Rely only on myself □ Rarely ask for help □ Sometimes ask for help □ Often seek help from family/friends/organizations

10.Regarding participation in group/organizational activities (e.g., political, religious, union):
□ Never □ Occasionally □ Frequently □ Actively and enthusiastically

### *****Utrecht Work Engagement Scale (UWES)*****

Instructions: The following questions concern your current work status. Please answer honestly. There are no right or wrong answers.

| **Item** | **Never (1)** | **Almost Never (2)** | **Rarely (3)** | **Sometimes (4)** | **Often (5)** | **Very Frequently (6)** | **Always (7)** |
| --- | --- | --- | --- | --- | --- | --- | --- |
| 1. At work, I feel bursting with energy. |  |  |  |  |  |  |  |
| 2. At work, I feel strong and vigorous. |  |  |  |  |  |  |  |
| 3. I am enthusiastic about my job. |  |  |  |  |  |  |  |
| 4. My job inspires me. |  |  |  |  |  |  |  |
| 5. When I get up in the morning, I look forward to going to work. |  |  |  |  |  |  |  |
| 6. I feel happy when I’m working intensely. |  |  |  |  |  |  |  |
| 7. I am proud of the work that I do. |  |  |  |  |  |  |  |
| 8. I am immersed in my work. |  |  |  |  |  |  |  |
| 9. I feel I am absorbed to the point of losing track of time when working. |  |  |  |  |  |  |  |
